# SIFTER search: a web server for accurate phylogeny-based protein function prediction

**DOI:** 10.1093/nar/gkv461

**Published:** 2015-05-15

**Authors:** Sayed M. Sahraeian, Kevin R. Luo, Steven E. Brenner

**Affiliations:** 1 Department of Plant and Microbial Biology, University of California, Berkeley, CA 94720, USA; 2 Department of Molecular and Cell Biology, University of California, Berkeley, CA 94720, USA; 3 Physical Biosciences Division, Lawrence Berkeley National Laboratory, Berkeley, CA 94720, USA

## Abstract

We are awash in proteins discovered through high-throughput sequencing projects. As only a minuscule fraction of these have been experimentally characterized, computational methods are widely used for automated annotation. Here, we introduce a user-friendly web interface for accurate protein function prediction using the SIFTER algorithm. SIFTER is a state-of-the-art sequence-based gene molecular function prediction algorithm that uses a statistical model of function evolution to incorporate annotations throughout the phylogenetic tree. Due to the resources needed by the SIFTER algorithm, running SIFTER locally is not trivial for most users, especially for large-scale problems. The SIFTER web server thus provides access to precomputed predictions on 16 863 537 proteins from 232 403 species. Users can explore SIFTER predictions with queries for proteins, species, functions, and homologs of sequences not in the precomputed prediction set. The SIFTER web server is accessible at http://sifter.berkeley.edu/ and the source code can be downloaded.

## INTRODUCTION

The accurate annotation of protein function is key to understanding life at the molecular level. With its inherent difficulty and expense, biochemical characterization of protein function cannot scale to accommodate the vast amount of sequence data already available, much less its continued growth. Thus, there is a need for reliable computational methods to predict protein function.

To computationally predict protein function, various schemes have been proposed so far using different data types such as sequence information (1–4), protein structure (5,6), phylogenetics and evolutionary relationships (7–10), interaction and association data (11–19) and a combination of these (20–26).

The traditional computational approach to predict function for an unknown protein transfers information from evolutionarily related proteins. Unfortunately, most such BLAST-motivated methods, which transfer the annotations from the most sequence-similar homologue, suffer from systematic flaws and thus have littered the databases with erroneous predictions. BLAST (27) is a sequence matching method, but sequence similarity does not directly reflect phylogeny (8) and may misrepresent the evolutionary structure of the tree in terms of the branching order and duplication/speciation events in the internal nodes.

SIFTER (Statistical Inference of Function Through Evolutionary Relationships) is a statistical approach for predicting protein molecular function that uses a protein family's phylogenetic tree as the natural structure for representing protein relationships (7,8). It overlays a phylogenetic tree with all known protein functions in the family and uses a statistical graphical model of function evolution to incorporate annotations throughout the tree. Predictions are supported by posterior probabilities for every protein in the family. SIFTER has been shown previously to perform better than other methods in widespread use.

The first Critical Assessment of Function Annotation (CAFA) experiment provided an independent assessment, and SIFTER was honored as a ‘top-performing method’ (28). Recently, SIFTER performed with distinction in the second CAFA experiment. In this experiment, SIFTER predicted function for nearly 100 000 sequences of unknown function, provided by the CAFA organizers. The organizers then assessed the ∼50 submitted methods, and their preliminary evaluations show that SIFTER is among the top four approaches overall in the molecular function category. Notably, in CAFA the improvement of SIFTER predictions over those from BLAST method is comparable to the improvement of BLAST over naïve weighted random prediction.

Open source code for SIFTER has been available since its first publication and remains so. It has been used by several other groups, and adapted for their own use (29). However, the data and CPU resources required for running SIFTER locally make this impractical for most users. For instance, running SIFTER for a protein with a domain in a large family may take several days to finish. The SIFTER web server thus provides access to results for users who do not wish to invest a local deployment. Because SIFTER naturally works on the whole family and since its running time may be longer than users are accustomed to waiting for BLAST results, we have precomputed the results on the entire set of families in the Pfam database version 27.0 (30) that have at least one experimentally annotated protein. This embodies 16 863 537 proteins from 232 403 species, precomputed with specially optimized parameters for SIFTER that were developed for this web site. Thus, this web server provides easy and rapid access to protein function predictions using a state-of-the-art sequence-based protein function prediction algorithm.

Users can access the protein function predictions by searching for one or multiple proteins (using UniProt identifiers or protein sequences), searching for all proteins in a given species, or searching for proteins in a given species that are predicted to have the given functions. For proteins not yet in our precomputed prediction set, users can submit the protein sequence and the web server will show the predictions for homologs of that gene in our prediction set (found based on top BLAST hits). The input field provides autocomplete and text suggestions to help users enter their queries. The server reports predictions based on experimental evidence alone by default for the highest precision. For more advanced predictions with broader coverage, users can combine other evidence. SIFTER then outputs predicted functions along with confidence scores for each function that reflect the reliability of the predictions.

Users can also download SIFTER code and run SIFTER locally. The database of the precomputed SIFTER results and associated query scripts are available for download as well.

## SIFTER APPROACH

**Figure 1.**
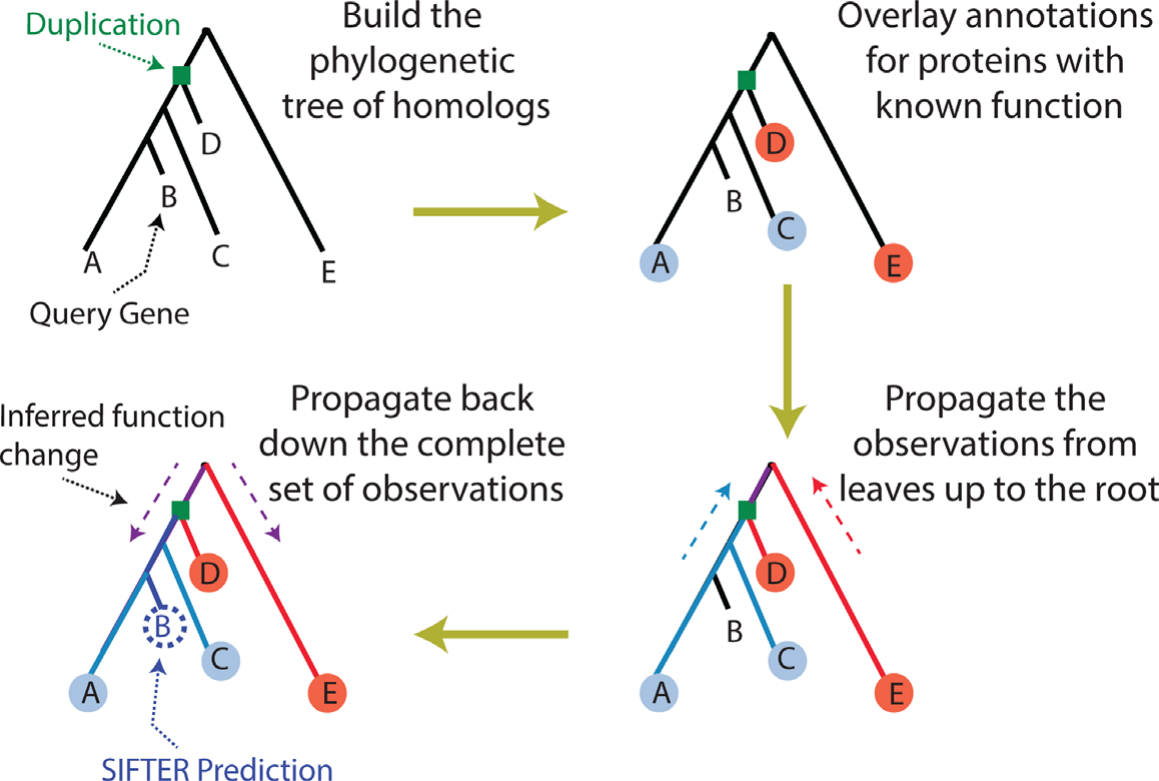
Phylogeny-based protein function prediction with SIFTER. The reconciliation distinguishes duplication from speciation at each internal node. Colors indicate functions.

Figure 1 illustrates the conceptual approach used in SIFTER for protein function prediction. SIFTER uses a statistical model of function evolution to incorporate annotations throughout the evolutionary tree. First, SIFTER takes the reconciled phylogenetic tree of homologs of the given query protein (protein B in Figure 1). Then it overlays known annotations of all homologous proteins on the tree. In the figure, proteins A and C are annotated with the blue function, while proteins D and E are annotated with the red function. SIFTER propagates the overlaid annotations up to the root using its underlying statistical model of function evolution. This model incorporates details such as branch length and distinguishes duplication from speciation events. It then propagates back down the complete set of information. Based on the propagated information, it infers the posterior probabilities of candidate functions for every protein in the family. That is, it infers for the query protein B the posterior probability of having either blue or red function. In this example, it is more likely that the tree has only one functional change (indicated by the arrow), in which the ancestral red function is mutated to the blue function on one lineage, following the duplication. So the blue function is a more likely annotation for the query protein. SIFTER as a phylogenetics-based approach reaches this conclusion naturally and the blue function will have higher posterior probability in SIFTER predictions for the query protein B.

SIFTER's phylogenetics-based approach to protein function prediction avoids systematic flaws in pairwise methods like BLAST. Lineage-specific rate variation is a complex phenomenon that may lead to a situation where the most similar sequences according to BLAST (i.e., roughly those with the shortest path length in the tree) may not be the sequences most recently diverged from the query sequence. For instance in the above example, BLAST will predict the red function due to the shortest path length from the query protein B to the protein D. Thus, the approach of using the most significant hits according to BLAST is systematically flawed and may yield erroneous results even as the number of known protein sequences increases. SIFTER incorporates the evolutionary history, minimizes problems due to rate variation and suggests an evolutionarily principled means of merging functional evidence from homologous proteins.

Another important factor in the SIFTER algorithm that sets it apart from some other techniques is that it systematically weighs the input evidence based on the quality of the annotations. As different experimental and computational functional annotation techniques have different error rates, uniformly treating the existing annotations in the databases as accurate propagates large numbers of erroneous annotations into predictions (31). For this reason, SIFTER incorporates evidence about annotation quality using a set of prior probabilities, which inform all analyses.

## SIFTER PERFORMANCE

**Figure 2.**
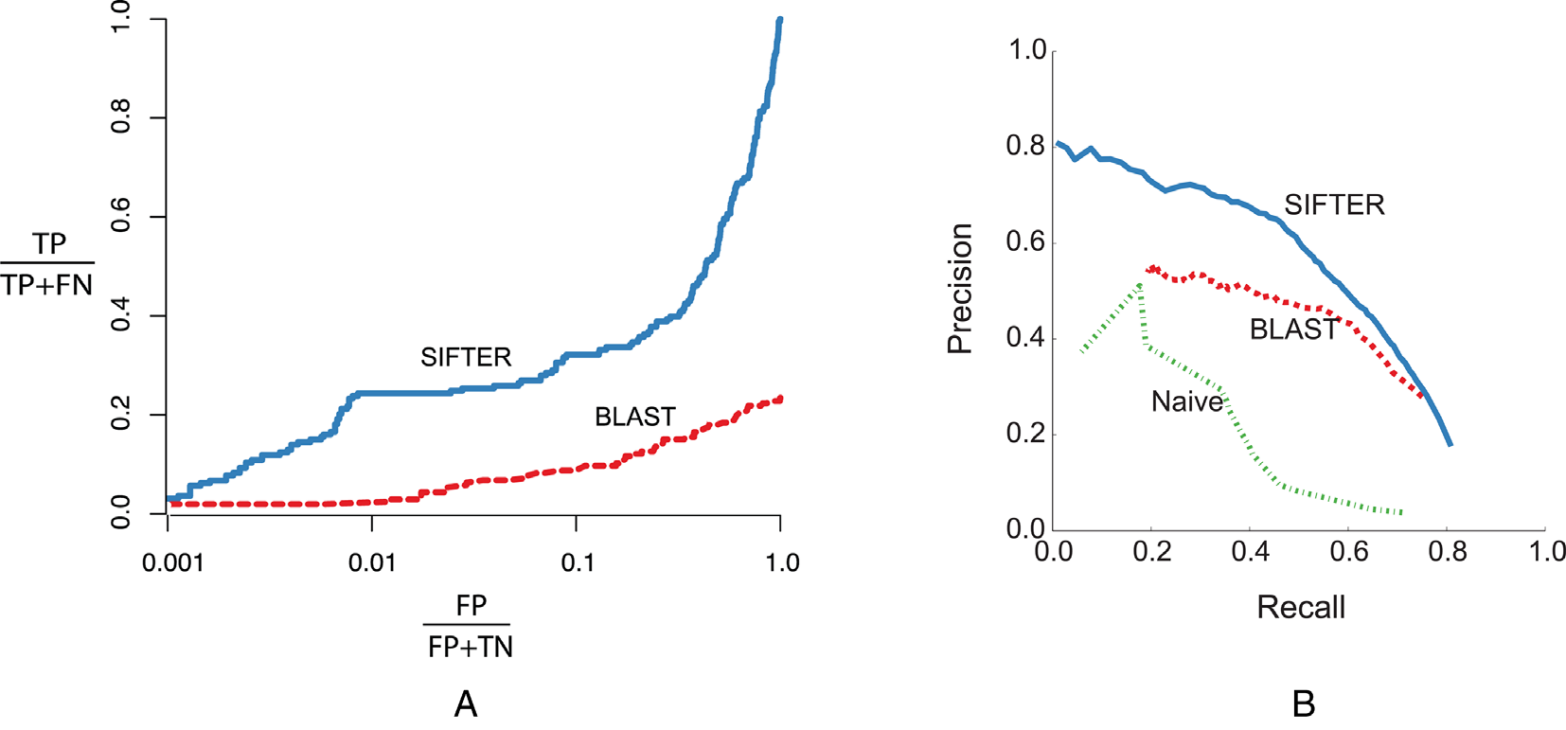
(**A**) The ROC-like comparison of SIFTER with BLAST for the Nudix family of proteins. SIFTER consistently dominates BLAST annotations in this family. (Figure adapted from (8)). (**B**) The CAFA precision-recall analysis of SIFTER, BLAST and naïve weighted random. (Data provided by CAFA2 analyst, Jiang Yuxiang).

SIFTER has been shown to outperform other sequence-based techniques in widespread use (7,8,28). Figure 2(a) compares SIFTER performance with BLAST for the Nudix family of proteins. Nudix is a difficult protein family to predict function for due to high sequence diversity that reduces the alignment quality of these proteins, which, in turn, reduces the quality of the phylogeny. In this figure, we see that SIFTER outperforms BLAST at all levels of false positives. At 99% specificity, we can observe that approximately 2.4% of annotations are correct in BLAST whereas 24.4% of the annotations are correct for SIFTER.

As an independent analysis of SIFTER performance, Figure 2(b) compares the precision-recall curve for SIFTER predictions against the BLAST predictions as well as the naive random predictions on CAFA2 benchmark genes. These are the 1139 genes out of 100 000 CAFA2 target genes that acquired experimental annotations during the waiting period of 6 months in the CAFA2 experiment. Here, naïve is a weighted random prediction in which every protein was ‘predicted’ to have functions proportional to the relative frequency of the terms in Swiss-Prot. As we see, the improvement of SIFTER over the BLAST method may be as great as the improvement of BLAST over naïve random prediction. For instance, at the recall rate of 30%, the precision rate of SIFTER, BLAST and naïve random predictions are respectively 72%, 53% and 32%.

## SIFTER SEARCH

The SIFTER web server provides easy access to SIFTER predictions of protein functions.

To assist users with different types of queries, the SIFTER web server is designed to provide search results for different input types. Here, we detail the search capabilities implemented in the server.

### Quick search

Users can access SIFTER predictions either through ‘Quick’ or ‘Advanced’ search. The Quick Search option is a single input field search mechanism that can identify different query types (including proteins, species and functions) and provide search results accordingly.

#### Input

Input to the quick search field can be any of the following:

- **Protein**: Users can search for the predicted functions for their query protein. The server accepts both UniProt accession number and UniProt ID (for one protein).
- **Species**: Users can search for the predicted functions for all proteins in a given species. The server accepts both species name and NCBI taxonomy identifier.
- **Function**: Users can search for proteins that are predicted to have the given functions in a given species. The server accepts both function name and Gene Ontology (GO) molecular function identifier.

The input field in the quick search option can provide auto-complete and text suggestions for the given input to help users enter their queries.

For any of the above query types, the SIFTER web server provides corresponding search results as detailed below.

#### Output

**Figure 3.**
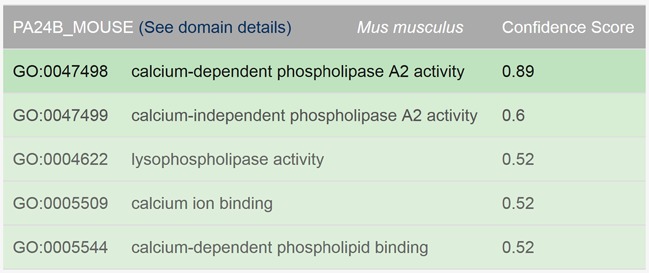
Sample output for searching SIFTER predictions by protein ID. Results are shown for protein PA24B_MOUSE.

SIFTER web server output is the set of predictions for the submitted query. The predicted functions are selected from the Gene Ontology molecular function ontology (32). The SIFTER web server provides the following outputs based on the query type it has identified as discussed above:

- **Protein**: If the entered protein identifier is in the precomputed results, the SIFTER web server provides the list of predicted functions from the GO molecular function ontology for the queried protein, along with a confidence score (between 0 and 1) for each function. This score is determined based on the number of candidate functions, the family size, and the frequency and distribution of candidate terms in the phylogenetic tree. Figure 3 shows sample output for a queried protein.
- **Species**: The web server provides the list of predicted functions for all of the proteins of the entered species that have predictions in the precomputed results.
- **Function**: The web server lists all the proteins in the queried species that have the entered function (or any of its descendants in the GO directed acyclic graph) as their top prediction.

### Advanced search

For more advanced predictions, users can pick their specific querying method from the list of four available options: ‘Predict by protein ID,’ ‘Predict for all proteins of a species,’ ‘Find proteins that have given functions,’ and ‘Predict for homologs of given sequences.’ The first three options are more advanced versions of the quick search discussed above. In the advanced search, users can search for multiple proteins or functions simultaneously using the ‘Predict by protein ID’ and ‘Find proteins that have given functions’ options. The last option in the advanced search, ‘Predict for homologs of given sequences,’ allows users to search for proteins not yet in our precomputed prediction set, as discussed below.

### Predictions for multi-domain proteins

SIFTER predicts function for each domain of a protein separately, using the phylogenetic tree of the family of each domain. To provide a single set of predictions for each protein, the SIFTER web server combines the individual domain predictions, assuming independence of domain functions. Consider protein *g* with *k* domains *g*_*i*_, where *i* = 1⋅⋅⋅*k*, and $s_{g_i}(f)$ is the probability that *i*^th^ domain has function *f*. We then compute *s*_*g*_(*f*), the probability that protein *g* has function *f*, as:

(1) \begin{equation*} s_g(f)=1-\prod _{i=1}^{k}(1-s_{g_i}(f)) \end{equation*}

The web server reports the combined *s*_*g*_(*f*) score as the default prediction for the query protein *g*. However, users may also access the predictions for each individual domain for more insight about domain-specific annotations. Users may also download the phylogenetic tree of each specific domain, overlaid with experimental annotations in the family.

#### Predict for homologs of given sequences

**Figure 4.**
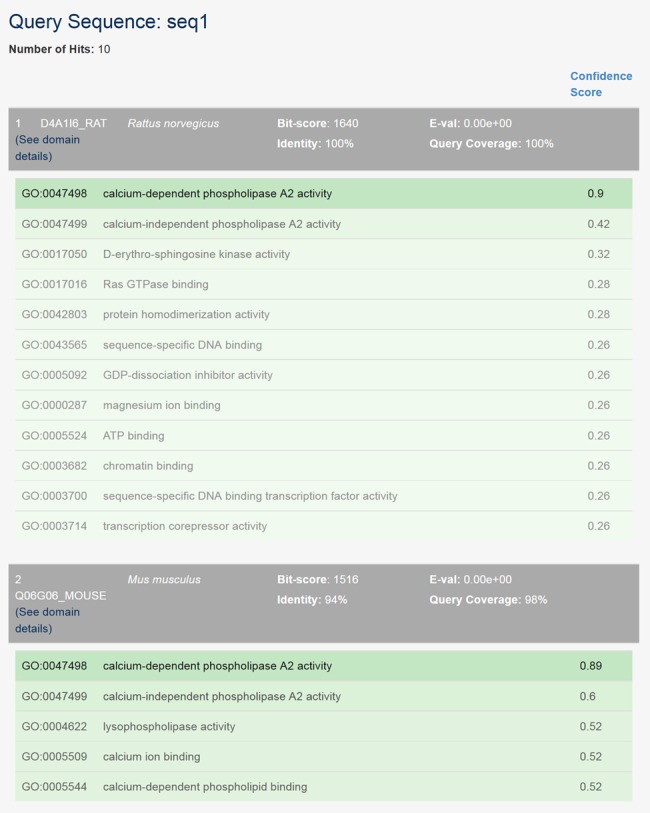
Sample output for searching SIFTER predictions for homologs of a given sequence.

In this option, users can submit the sequences of proteins not yet in our precomputed prediction set, and the web server will show the predictions for the closest homologs in our prediction set (found based on top BLAST hits obtained from the NCBI-BLAST website (33)). The output includes the statistics of the significance of the hits along with the SIFTER predictions for each of the hits to the given input sequence. For each search, users can enter up to 10 protein sequences. Figure 4 illustrates sample output for a queried sequence. While this option embodies limitations of BLAST, often there is such a close homolog with a precomputed SIFTER prediction that the errors are limited. By contrast, the distance to the closest experimentally annotated protein is often so far as to introduce significant errors.

#### More control on the SIFTER options in Advanced Search

For highest precision, the server reports predictions based on experimental evidence by default. However, in Advanced Search, users can access more predictions for special applications by combining other lower quality but more extensive evidence. Users can also balance the sensitivity/specificity trade-off by adjusting the relative weighting of the SIFTER predictions obtained based exclusively on experimental evidence versus those predictions that include non-experimental evidence. As the default, for advanced combined predictions we use the weighting scheme that performed well in the second CAFA experiment.

### Estimating SIFTER processing time on a local machine

**Figure 5.**
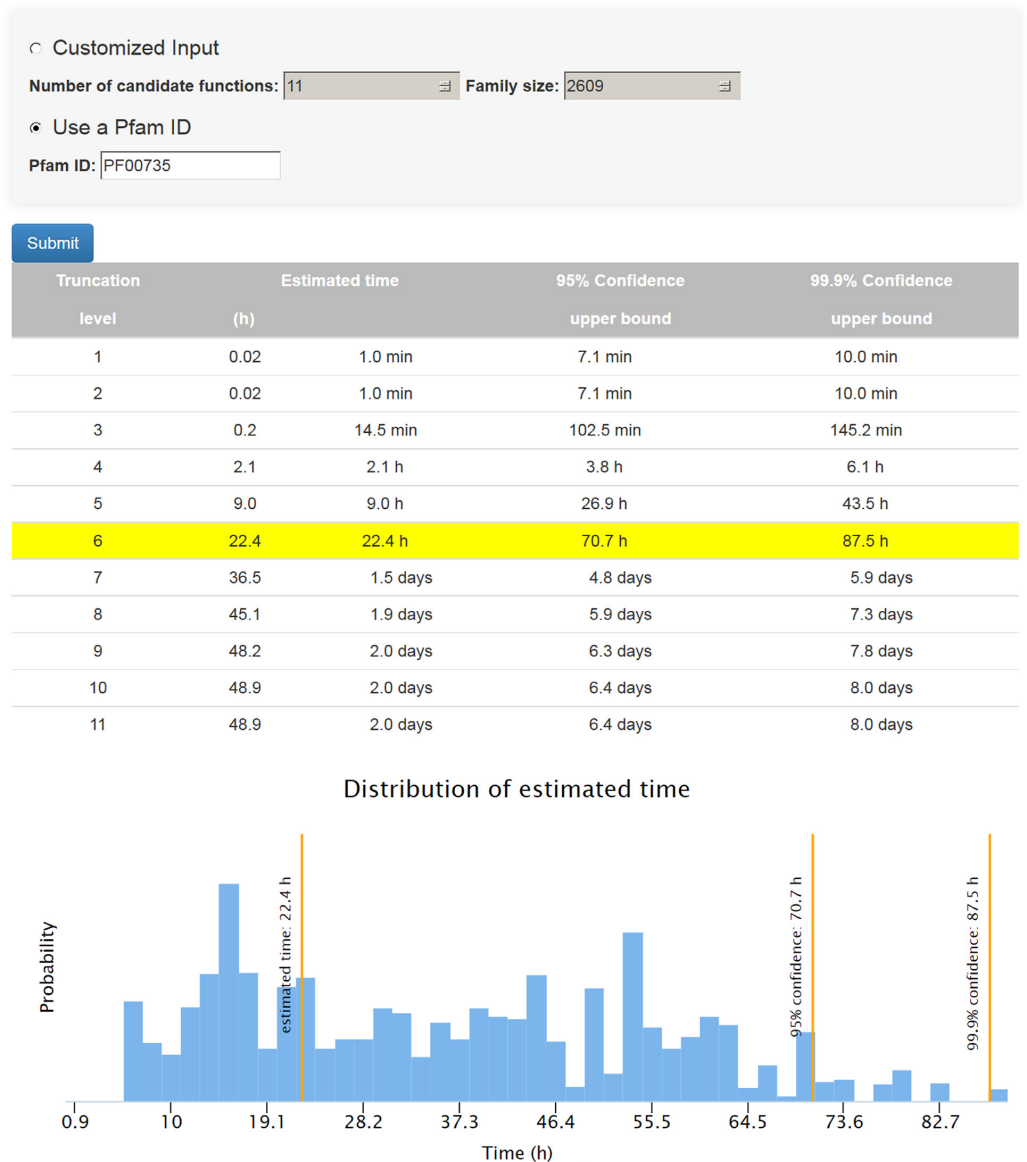
Estimating SIFTER processing time for a sample Pfam family (PF00735) with 11 candidate molecular functions and family size 2609.

Users can download the SIFTER source code and run it locally. Since the time complexity of the algorithm underlying SIFTER is exponential in the number of candidate molecular functions, SIFTER offers a heuristic that sets the maximum number of molecular functions present in any protein. This significantly reduces the running time with minimal impact on the prediction results (8). In order to select the truncation level that maximizes prediction accuracy without exceeding the available running time, users can estimate the processing time of a given family for each heuristic level. In the SIFTER web server, we provide an interactive framework that yields this estimate using a predictive model trained on existing SIFTER data. Users can enter the Pfam ID of their family of interest or provide the number of candidate molecular functions and family size of their query. Figure 5 illustrates complexity estimations for a sample protein family.

### Data sources and updates

To precompute the SIFTER web server predictions, we used the UniProt Gene Ontology Annotation (GOA) database (34) (update 09/2013) as the source of annotation. SIFTER currently uses the Gene Ontology (32) molecular function ontology (update 09/2013). Pfam v27.0 (30) provides protein families used to build the phylogenetic trees. We downloaded aligned homologous protein domain sequences from Pfam, and generated the phylogenetic tree using FastTree version 2.1.3 (35) with default parameters. We divided large families into smaller sub-trees and ran SIFTER on each sub-tree. For such families we also ran SIFTER with the truncation heuristic parameter 1 (8), and we merged these results to obtain final predictions. We will update all predictions with any major release of the Pfam database and also annually to include new annotations in GOA.

## DISCUSSION

SIFTER is a state-of-the-art sequence-based protein function prediction algorithm that infers protein function using a statistical model of function evolution in the phylogenetic tree. Here, we introduced the SIFTER web server, which provides easy access to precomputed SIFTER predictions using specially optimized parameters. Users can search SIFTER predictions using different querying schemes such as querying for a protein, all proteins of a species, or proteins predicted to have a function. The web server also provides predictions for homologs of proteins not yet in our precomputed prediction set. Thus, the SIFTER web server provides a unique environment to explore the SIFTER function prediction algorithm easily and swiftly.

## References

[1] Wass M.N., Sternberg M.J.E. (2008). ConFunc-functional annotation in the twilight zone. Bioinformatics.

[2] Martin D.M., Berriman M., Barton G.J. (2004). GOtcha: a new method for prediction of protein function assessed by the annotation of seven genomes. BMC Bioinformatics.

[3] Hawkins T., Luban S., Kihara D. (2006). Enhanced automated function prediction using distantly related sequences and contextual association by PFP. Protein Sci..

[4] Clark W.T., Radivojac P. (2011). Analysis of protein function and its prediction from amino acid sequence. Proteins.

[5] Pazos F., Sternberg M.J. (2004). Automated prediction of protein function and detection of functional sites from structure. Proc. Natl. Acad. Sci. U.S.A..

[6] Pal D., Eisenberg D. (2005). Inference of protein function from protein structure. Structure.

[7] Engelhardt B.E., Jordan M.I., Muratore K.E., Brenner S.E. (2005). Protein molecular function prediction by Bayesian phylogenomics. PLoS Comput. Biol..

[8] Engelhardt B.E., Jordan M.I., Srouji J.R., Brenner S.E. (2011). Genome-scale phylogenetic function annotation of large and diverse protein families. Genome Res..

[9] Gaudet P., Livstone M.S., Lewis S.E., Thomas P.D. (2011). Phylogenetic-based propagation of functional annotations within the Gene Ontology consortium. Brief. Bioinformatics.

[10] Eisen J.A. (1998). Phylogenomics: improving functional predictions for uncharacterized genes by evolutionary analysis. Genome Res..

[11] Chua H.N., Sung W.-K., Wong L. (2006). Exploiting indirect neighbours and topological weight to predict protein function from protein-protein interactions. Bioinformatics.

[12] Vazquez A., Flammini A., Maritan A., Vespignani A. (2003). Global protein function prediction from protein-protein interaction networks. Nat. Biotechnol..

[13] Deng M., Zhang K., Mehta S., Chen T., Sun F. (2003). Prediction of protein function using protein-protein interaction data. J. Comput. Biol..

[14] Letovsky S., Kasif S. (2003). Predicting protein function from protein-protein interaction data: a probabilistic approach. Bioinformatics.

[15] Nabieva E., Jim K., Agarwal A., Chazelle B., Singh M. (2005). Whole-proteome prediction of protein function via graph-theoretic analysis of interaction maps. Bioinformatics.

[16] Sharan R., Ulitsky I., Shamir R. (2007). Network-based prediction of protein function. Mol. Syst. Biol..

[17] Mostafavi S., Morris Q. (2012). Combining many interaction networks to predict gene function and analyze gene lists. Proteomics.

[18] Hulsman M., Dimitrakopoulos C., de Ridder J.D. (2014). Scale-space measures for graph topology link protein network architecture to function. Bioinformatics.

[19] Cao M., Pietras C.M., Feng X., Doroschak K.J., Schaffner T., Park J., Zhang H., Cowen L.J., Hescott B.J. (2014). New directions for diffusion-based network prediction of protein function: incorporating pathways with confidence. Bioinformatics.

[20] Guan Y., Myers C.L., Hess D.C., Barutcuoglu Z., Caudy A.A., Troyanskaya O.G. (2008). Predicting gene function in a hierarchical context with an ensemble of classifiers. Genome Biol..

[21] Troyanskaya O.G., Dolinski K., Owen A.B., Altman R.B., Botstein D. (2003). A Bayesian framework for combining heterogeneous data sources for gene function prediction (in Saccharomyces cerevisiae). Proc. Natl. Acad. Sci. U.S.A..

[22] Obozinski G., Lanckriet G., Grant C., Jordan M.I., Noble W.S. (2008). Consistent probabilistic outputs for protein function prediction. Genome Biol..

[23] Costello J.C., Dalkilic M.M., Beason S.M., Gehlhausen J.R., Patwardhan R., Middha S., Eads B.D., Andrews J.R. (2009). Gene networks in Drosophila melanogaster: integrating experimental data to predict gene function. Genome Biol..

[24] Kourmpetis Y.A., van Dijk A.D., Bink M.C., van Ham R.C., ter Braak C.J. (2010). Bayesian Markov Random Field analysis for protein function prediction based on network data. PLoS One.

[25] Wass M.N., Barton G., Sternberg M. J.E. (2012). CombFunc: predicting protein function using heterogeneous data sources. Nucleic Acids Res..

[26] Lee I., Date S.V., Adai A.T., Marcotte E.M. (2004). A probabilistic functional network of yeast genes. Science.

[27] Altschul S.F., Gish W., Miller W., Myers E.W., Lipman D.J. (1990). Basic local alignment search tool. J. Mol. Biol..

[28] Radivojac P., Clark W.T., Oron T.R., Schnoes A.M., Wittkop T., Sokolov A., Graim K., Funk C., Verspoor K., Ben-Hur A. (2013). A large-scale evaluation of computational protein function prediction. Nat. Methods.

[29] Jocker A., Hoffmann F., Groscurth A., Schoof H. (2008). Protein function prediction and annotation in an integrated environment powered by web services (AFAWE). Bioinformatics.

[30] Finn R.D., Bateman A., Clements J., Coggill P., Eberhardt R.Y., Eddy S.R., Heger A., Hetherington K., Holm L., Mistry J. (2014). Pfam: the protein families database. Nucleic Acids Res..

[31] Brenner S.E. (1999). Errors in genome annotation. Trends Genet..

[32] Ashburner M., Ball C.A., Blake J.A., Botstein D., Butler H., Cherry J.M., Davis A.P., Dolinski K., Dwight S.S., Eppig J.T. (2000). Gene ontology: tool for the unification of biology. The Gene Ontology Consortium. Nat. Genet..

[33] Boratyn G.M., Camacho C., Cooper P.S., Coulouris G., Fong A., Ma N., Madden T.L., Matten W.T., McGinnis S.D., Merezhuk Y. (2013). BLAST: a more efficient report with usability improvements. Nucleic Acids Res..

[34] Barrell D., Dimmer E., Huntley R.P., Binns D., O'Donovan C., Apweiler R. (2009). The GOA database in 2009–an integrated Gene Ontology Annotation resource. Nucleic Acids Res..

[35] Price M.N., Dehal P.S., Arkin A.P. (2009). FastTree: computing large minimum evolution trees with profiles instead of a distance matrix. Mol. Biol. Evol..

